# Cytotoxic Mediators in Paradoxical HIV–Tuberculosis Immune Reconstitution Inflammatory Syndrome

**DOI:** 10.4049/jimmunol.1402105

**Published:** 2015-01-14

**Authors:** Katalin A. Wilkinson, Naomi F. Walker, Graeme Meintjes, Armin Deffur, Mark P. Nicol, Keira H. Skolimowska, Kerryn Matthews, Rebecca Tadokera, Ronnett Seldon, Gary Maartens, Molebogeng X. Rangaka, Gurdyal S. Besra, Robert J. Wilkinson

**Affiliations:** * Clinical Infectious Diseases Research Initiative, University of Cape Town, Cape Town, 7925 South Africa;; † Department of Medicine, University of Cape Town, Cape Town, 7925 South Africa;; ‡ Medical Research Council National Institute for Medical Research, London NW7 1AA, United Kingdom;; § Division of Medicine, Imperial College London, London W2 1PG, United Kingdom;; ¶ Division of Medical Microbiology, University of Cape Town, Cape Town, 7925 South Africa;; ‖ National Health Laboratory Service, Cape Town, 7925 South Africa; and; # School of Biosciences, University of Birmingham, Birmingham B15 2TT, United Kingdom

## Abstract

Tuberculosis-associated immune reconstitution inflammatory syndrome (TB-IRIS) frequently complicates combined antiretroviral therapy and antituberculosis therapy in HIV-1–coinfected tuberculosis patients. The immunopathological mechanisms underlying TB-IRIS are incompletely defined, and improved understanding is required to derive new treatments and to reduce associated morbidity and mortality. We performed longitudinal and cross-sectional analyses of human PBMCs from paradoxical TB-IRIS patients and non-IRIS controls (HIV-TB–coinfected patients commencing antiretroviral therapy who did not develop TB-IRIS). Freshly isolated PBMC stimulated with heat-killed *Mycobacterium tuberculosis* H37Rv (hkH37Rv) were used for IFN-γ ELISPOT and RNA extraction. Stored RNA was used for microarray and RT-PCR, whereas corresponding stored culture supernatants were used for ELISA. Stored PBMC were used for perforin and granzyme B ELISPOT and flow cytometry. There were significantly increased IFN-γ responses to hkH37Rv in TB-IRIS, compared with non-IRIS PBMC (*p* = 0.035). Microarray analysis of hkH37Rv-stimulated PBMC indicated that perforin 1 was the most significantly upregulated gene, with granzyme B among the top five (log_2_ fold difference 3.587 and 2.828, respectively), in TB-IRIS. Downstream experiments using RT-PCR, ELISA, and ELISPOT confirmed the increased expression and secretion of perforin and granzyme B. Moreover, granzyme B secretion reduced in PBMC from TB-IRIS patients during corticosteroid treatment. Invariant NKT cell (CD3^+^Vα24^+^) proportions were higher in TB-IRIS patients (*p* = 0.004) and were a source of perforin. Our data implicate the granule exocytosis pathway in TB-IRIS pathophysiology. Further understanding of the immunopathogenesis of this condition will facilitate development of specific diagnostic and improved therapeutic options.

## Introduction

Human immunodeficiency virus-1 is recognized as the strongest predisposing factor to tuberculosis (TB), and TB is the commonest cause of death in HIV-1–infected persons in Africa (1, 2). However, otherwise beneficial dual therapy for HIV-1 and TB is frequently complicated by the occurrence of the TB-associated immune reconstitution inflammatory syndrome (TB-IRIS), an early complication of combination antiretroviral therapy (ART).

Two forms of TB-IRIS are recognized: paradoxical, which occurs in patients established on antituberculosis therapy before ART, but who develop recurrent or new TB symptoms and clinical features after ART initiation; and unmasking TB-IRIS in patients not receiving treatment for TB when ART is started, but who present with active TB within 3 mo of starting ART (3). Paradoxical TB-IRIS affects ∼15.9% of all HIV-1–infected patients commencing ART while on TB treatment, and up to 54% in some populations, causing considerable morbidity and mortality (4, 5). Immunosuppressive corticosteroid therapy improves symptoms and reduces hospital admissions, but is not without adverse events, and is potentially detrimental in cases of drug-resistant TB (6–8). Specific diagnostic tools and treatments for TB-IRIS are lacking, and understanding the pathogenesis of this condition is important to assist in the development of more specific therapies.

Risk factors for TB-IRIS, such as low CD4 count and disseminated TB disease at presentation, suggest that a pathological immune reaction to mycobacterial Ags during immune recovery is responsible. We previously described highly dynamic Ag-specific CD4 T cell IFN-γ responses in the first weeks after ART initiation in both TB-IRIS and control patients in response to early secretory antigenic target-6, 38-kDa cell wall–associated Ag, and α-crystallins 1 and 2 (9). However, such PBMC Th1 expansions to recombinant protein Ags of *Mycobacterium tuberculosis* were common to both TB-IRIS patients and controls. We have subsequently shown a role for hypercytokinaemia, of predominantly myeloid or dual myeloid/lymphoid origin in TB-IRIS as well as matrix metalloproteinase dysregulation (10, 11). Moreover, the beneficial effects of prednisone in TB-IRIS appear to be associated with suppression of proinflammatory cytokine responses of innate immune origin (8, 12), suggesting that innate immune responses may have a role in TB-IRIS pathophysiology.

In the current study, we compared the immune responses in TB-IRIS patients with non-IRIS controls after restimulation with heat-killed (hk) whole *M. tuberculosis* bacillus (using the H37Rv laboratory strain), which contains a wide range of both protein and nonprotein Ags. We found that restimulation with hkH37Rv resulted in an increased IFN-γ release by TB-IRIS PBMC, raising the possibility that a component of the T cell response is directed toward nonprotein Ags and may be responsible for the differential response. Unbiased analysis of hkH37Rv-stimulated PBMC by microarray indicated increased abundance of transcripts for granzyme B and perforin in TB-IRIS patients. Our downstream RT-PCR, ELISA, and ELISPOT analyses confirmed increased expression as well as secretion, implicating the involvement of the granule exocytosis pathway in TB-IRIS pathophysiology. A subset of PBMC expressing both CD3 and the Vα24 chain of the TCR, indicative of invariant NKT (iNKT) cells, was increased in TB-IRIS patients and contributed to perforin production. Our data support the hypothesis that the granule exocytosis pathway plays a role in TB-IRIS pathophysiology, and further study of this pathway may elucidate novel therapeutic targets in TB-IRIS.

## Materials and Methods

### Participants

The University of Cape Town Faculty of Health Sciences Human Research Ethics Committee (HREC references 337/2004, 173/2005) approved the study. Participants provided written informed consent. Blood samples were collected continuously and prospectively between March 2005 and December 2007 at Ubuntu Clinic, Site B Khayelitsha and G.F. Jooste Hospital. Previous cross-sectional and longitudinal analyses of patients from this cohort, including a randomized controlled trial of prednisone versus placebo in TB-IRIS patients, have been reported (7, 9, 10). Active TB was diagnosed on the basis of smear or culture positivity, or in cases of smear-negative TB, according to international guidelines (6, 10, 13). Patients were started on first-line antituberculosis therapy according to national guidelines. All patients were ART naive at enrollment. First-line ART at the time of the study was most often stavudine, lamivudine, and efavirenz. The diagnosis of TB-IRIS was made according to a consensus case definition that has been independently validated (3, 14, 15). Patients were followed up at regular intervals for at least 2 mo post-ART initiation, and those who did not develop TB-IRIS were designated non-IRIS controls.

The current analyses included 62 patients with TB-IRIS (38 female, 24 male, median age 31 y, median baseline CD4 count 57, median number of days to development of IRIS symptoms was 14) and 34 non-IRIS patients (25 female, 9 male, median age 35 y, median baseline CD4 count 50) (Supplemental Table I). Among the 62 TB-IRIS patients, active TB was diagnosed on the basis of smear or culture positivity in 51 (82.5%), or according to international guidelines in cases of smear-negative TB (*n* = 11, 17.5%). Of the non-IRIS controls, 18 patients had microbiological confirmation of TB. No significant differences were found between IRIS and non-IRIS patients regarding gender, baseline CD4 T cell counts, or age.

### Sample collection and processing

Venous blood collected in sodium heparin vacutainers (BD Pharmingen) was processed within 4 h of collection. PBMC were isolated by density-gradient centrifugation over Ficoll. Freshly isolated PBMC were stimulated with hkH37Rv and used for RNA extraction (as described below), as well as measurement of IFN-γ release by ELISPOT analysis, whereas remaining PBMC were cryopreserved in temperature-monitored liquid nitrogen tanks.

### ELISPOT analysis

IFN-γ ELISPOT was performed using fresh PBMC, as previously described (16, 17). Perforin and granzyme B ELISPOTs were performed using stored PBMC (following overnight resting) and the human granzyme B and human perforin ELISPOT kits (Mabtech AB), following the manufacturer’s recommendations. Briefly, to detect human granzyme B, polyvinylidene difluoride ELISPOT plates were prewet with 50 μl 70% ethanol for 2 min, followed by washing five times with sterile water at 200 μl/well. A total of 15 μg/ml coating Ab (GB10) was added in sterile PBS at 100 μl/well. After overnight incubation at 4°C, the plate was washed five times with PBS, and wells were blocked using RPMI 1640/10% FCS for 30 min at room temperature. Following the removal of the blocking medium, cells were added at 250,000 per well in 100 μl RPMI 1640/10% FCS, together with hkH37Rv at multiplicity of infection (MOI) = 1:1, H37Rv to PBMC, or left unstimulated. The plate was incubated overnight in the CO_2_ incubator at 37°C. The following morning, the cells were removed and the wells were washed five times with PBS. A total of 1 μg/ml detection Ab (GB11-biotin) was added in PBS containing 0.5% FCS at 100 μl/well for 2 h at room temperature. The wells were washed, and diluted streptavidin–alkaline phosphatase (1:1000) in PBS–0.5% FCS was added at 100 μl/well for 1 h at room temperature. Following a final wash, the wells were developed using the ready-to-use substrate solution 5-bromo-4-chloro-3-indolyl phosphate/NBT-plus, and color development was stopped using extensive washing with tap water. To detect human perforin-secreting cells, mAb Pf-80/164-precoated ELISPOT plates were used, which were washed four times with sterile PBS, followed by blocking with RPMI 1640/10% FCS for 30 min at room temperature. Cells were added at 250,000 per well in 100 μl RPMI 1640/10% FCS, together with hkH37Rv at MOI = 1:1, H37Rv to PBMC, or left unstimulated as controls. The plate was incubated in the CO_2_ incubator at 37°C for 24 h. Following removal of the cells, wells were washed five times with PBS, and the detection Ab (Pf-344-biotin), diluted to 1 μg/ml in PBS–0.5% FCS, was added at 100 μl/well for 2 h at room temperature. Wells were washed again, and streptavidin–alkaline phosphatase, diluted 1:1000 in PBS–0.5% FCS, was added at 100 μl/well for 1 h at room temperature. The wells were developed, as above. Results were counted using an AID ELISPOT reader (AID GmbH Germany, equipped with software version 5), and are reported as spot-forming cells per million/10^6^ PBMC with the unstimulated backgrounds subtracted. A positive response was defined as >30 spot-forming cells per million/10^6^ PBMC. All results were checked for data consistency and plausibility as per Minimal Information About T Cell Assays reporting requirements.

### RNA and protein secretion assays

For transcriptomic analysis, fresh PBMC were plated at a density of 1 × 10^6^ cells/ml, 5 ml per well in 6-well plates, rested overnight at 37°C, and then stimulated with hkH37Rv (MOI = 1:1, H37Rv to PBMC) for 6 or 24 h, or remained unstimulated. Cells were then lysed in cold RLN buffer (RNeasy mini kit for total RNA isolation; Qiagen, Valencia, CA). RNA was extracted using RNeasy Mini Kit Spin protocol as per the manufacturer’s instructions (Qiagen), and used for quantitative RT-PCR, as described (8, 10). Fold induction over unstimulated cultures was calculated by the ΔΔ cycle threshold method, and fold values log transformed to normalize. For RT-PCR, β-actin was used to normalize values. For protein secretion assays, cell culture supernatants were harvested at 24-h stimulation and stored at −80°C until measurement of granzyme A and B by ELISA (Bender MedSystems, Vienna, Austria), following the manufacturer’s protocol.

### Microarray analysis

Samples consisted of RNA extracted from PBMC stimulated with hkH37Rv, as described above, from seven TB-IRIS and seven control samples, matched by clinical data (age, sex, duration of antituberculosis therapy, and CD4 count), with one condition per patient (stimulated samples only) at a single time point (6 h). Samples were hybridized to an Affymetrix U133^+^ GeneChip, following standard procedures. Raw data files were processed using the PLIER algorithm, which incorporates background correction (ArrayAssist Lite; Stratagene, Cedar Creek, TX). Normalized data were log2 transformed; IRIS and non-IRIS samples were paired on the basis of clinical data and analyzed using Significance Analysis of Microarrays 3.0 (18), using the following parameters: seed for random number generator = 1234567, log2 scale = TRUE, median centering of array data = TRUE, analysis type = “Two class paired,” number of permutations = 200, and false discovery rate = 0.000001. In compliance with Minimum Information About a Microarray Experiment, the data were deposited in the National Center for Biotechnology Information’s Gene Expression Omnibus repository, with accession number GSE48237 (http://www.ncbi.nlm.nih.gov/geo/query/acc.cgi?acc=GSE48237).

### Flow cytometry

Flow cytometric analysis was performed using cryopreserved PBMC. Viability was ascertained by trypan blue exclusion. Cells were washed, and then stained on ice for 20 min with the following fluorescent Abs in various combinations: CD3 (PerCP-Cy5.5 or allophycocyanin) or FITC, anti–TCR-αβ-1-FITC, and anti–TCR-γδ-1-PE (all BD Oncomark); Vα24 TCR-FITC and Vβ11 TCR-PE (Immunotech); CD56-PE, CD107a-PE, CD94-allophycocyanin, CD158b-PE, and CD16–PerCP-Cy5.5 (all from BD Pharmingen); and CD158a–PerCP-Cy5.5 (eBiosciences). After washing, stained cells were fixed in PBS/2% FCS/1.6% paraformaldehyde and acquired on a FACSCalibur flow cytometer (BD Biosciences). For intracellular staining, cells were first surface stained, followed by washing and incubation for 30 min on ice with Fix/Perm buffer (eBioscience). After washing in permeabilization buffer, cells were incubated for 30 min on ice with perforin-PE (Perforin reagent set; BD Pharmingen 556437) or IFN-γ allophycocyanin. The cells were washed again, fixed in PBS/2% FCS/1.6% paraformaldehyde, and acquired. Data were analyzed using Flowjo software (Tree Star, Ashland, OR).

### Statistical analysis

Statistical analysis was performed using GraphPad Prism. The normality of data was assessed by the D’Agostino and Pearson omnibus normality test. Medians are quoted with the interquartile range (IQR). Paired parametric data were analyzed by Student paired *t* test, and nonparametric paired data were analyzed by Wilcoxon matched-pairs test. Unpaired parametric variables were assessed by Student unpaired *t* test, and nonparametric variables were assessed by Mann–Whitney *U* test. Significance was inferred from a *p* value <0.05.

## Results

### Differential IFN-γ response to hkH37Rv in TB-IRIS patients

**FIGURE 1.**
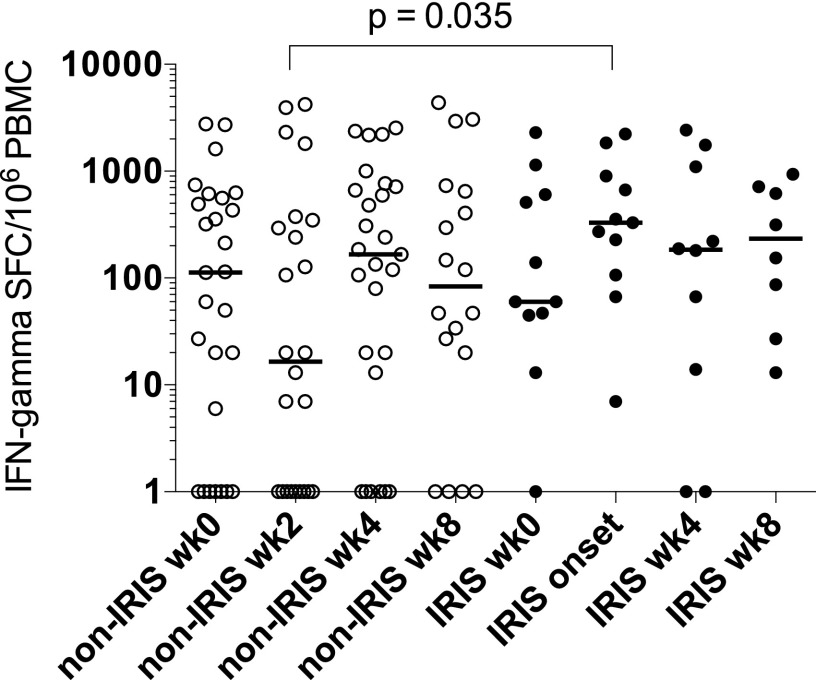
IFN-γ responses to hkH37Rv in TB-IRIS compared with non-IRIS control PBMC during the first 8 wk post-ART initiation. IFN-γ responses of PBMC measured by ELISPOT from 38 HIV-TB–coinfected patients at baseline and for 8 wk post-ART initiation, after stimulation with hkH37Rv. Eleven patients developed TB-IRIS at a median of 14 d. Significantly increased numbers of IFN-γ spot-forming cells (SFC) were identified in TB-IRIS patients at TB-IRIS onset, compared with non-IRIS control patients sampled at 2 wk of ART (*p* = 0.035). No differences in IFN-γ SFC were identified at other time points. Horizontal lines indicate median values.

We investigated the IFN-γ response to hkH37Rv in PBMC obtained from a prospective cohort of 38 HIV-TB–coinfected patients at baseline and 2-wk interval post-ART initiation. The clinical characteristics of the patients are shown in Supplemental Table I and have been previously described (9). Of the 38 TB patients, 11 developed TB-IRIS at a median of 2 wk (14 d, IQR 11–18 d) of ART. At this time, the median number of IFN-γ spot-forming cells per 10^6^ PBMC was 329 (IQR 107–905) in TB-IRIS patients, compared with 16.5 (IQR 0–336) in non-IRIS controls (*p* = 0.035; Fig. 1). There was no significant difference between IFN-γ responses at baseline, or weeks 4 and 8 post-ART initiation. These results indicate differentially increased IFN-γ release by TB-IRIS PBMC at the time of IRIS, which contrasts with our previous observations in response to recombinant protein Ags (9). Because hkH37Rv contains a wide range of protein and nonprotein Ags, these results raise the possibility that a component of the T cell response in TB-IRIS patients may be directed toward nonprotein Ags and may be responsible for the differential response.

### Increased transcript abundance for granzyme B and perforin in TB-IRIS PBMC

**FIGURE 2.**
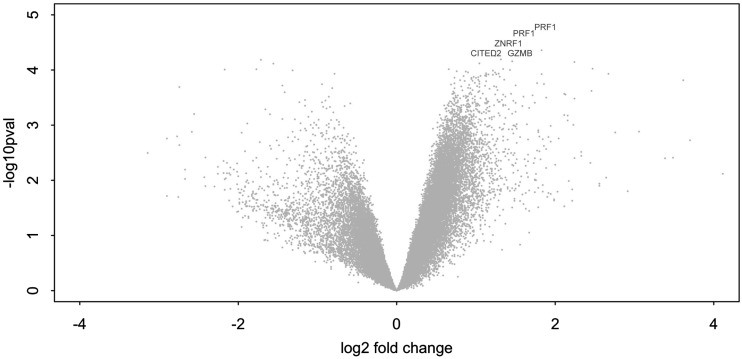
Representation of significantly differentially expressed genes. Microarray analysis of RNA from seven matched pairs of IRIS and non-IRIS patients, at 2 wk on ART, showed transcript for perforin and granzyme B genes to be among the four genes most significantly overrepresented in TB-IRIS PBMC after stimulation with H37Rv. The transcripts for Zinc and ring finger 1 and Cbp/p300-interacting transactivator with Glu/Asp-rich C-terminal domain 2 were also significantly raised. The genes significantly upregulated in TB-IRIS (with δ = 0.96 and false discovery rate of 0.000001%) are represented on the volcano plot as labeled. The *x*-axis shows the log2-fold change, and the *y*-axis shows a measure of significance (−log10 [*p* value]).

To explore differences between stimulated PBMC from patients with TB-IRIS as compared with non-IRIS patients that would account for the differentially increased IFN-γ release by TB-IRIS PBMC in response to hkH37Rv shown above, we investigated differential transcript abundance by microarray analysis of RNA from PBMC stimulated for 6 h with hkH37Rv. Seven TB-IRIS patients were compared with seven non-IRIS controls. RNA from unstimulated PBMC was not included as we wished to compare hkH37Rv stimulation-specific TB-IRIS gene expression with similarly stimulated non-IRIS controls. Clinical characteristics of the patients are shown in Supplemental Table I. The most significantly upregulated gene at 2 wk post-ART initiation (the median time of TB-IRIS onset) was perforin 1 identified by two individual probes for PRF1 gene (median log_2_ fold change = 3.587 and 3.092, respectively). Granzyme B was among the top five most significantly upregulated genes in TB-IRIS (median log_2_ fold change = 2.828), with δ = 0.96 and false discovery rate of 0% (Fig. 2, and data deposited GSE48237). Thus, unbiased analysis by microarray suggested the involvement of the granule exocytosis pathway in TB-IRIS at the time of IRIS, and we proceeded to downstream validate these findings for perforin and granzyme B.

### Secondary validation of microarray analysis by RT-PCR, ELISA, and ELISPOT

**FIGURE 3.**
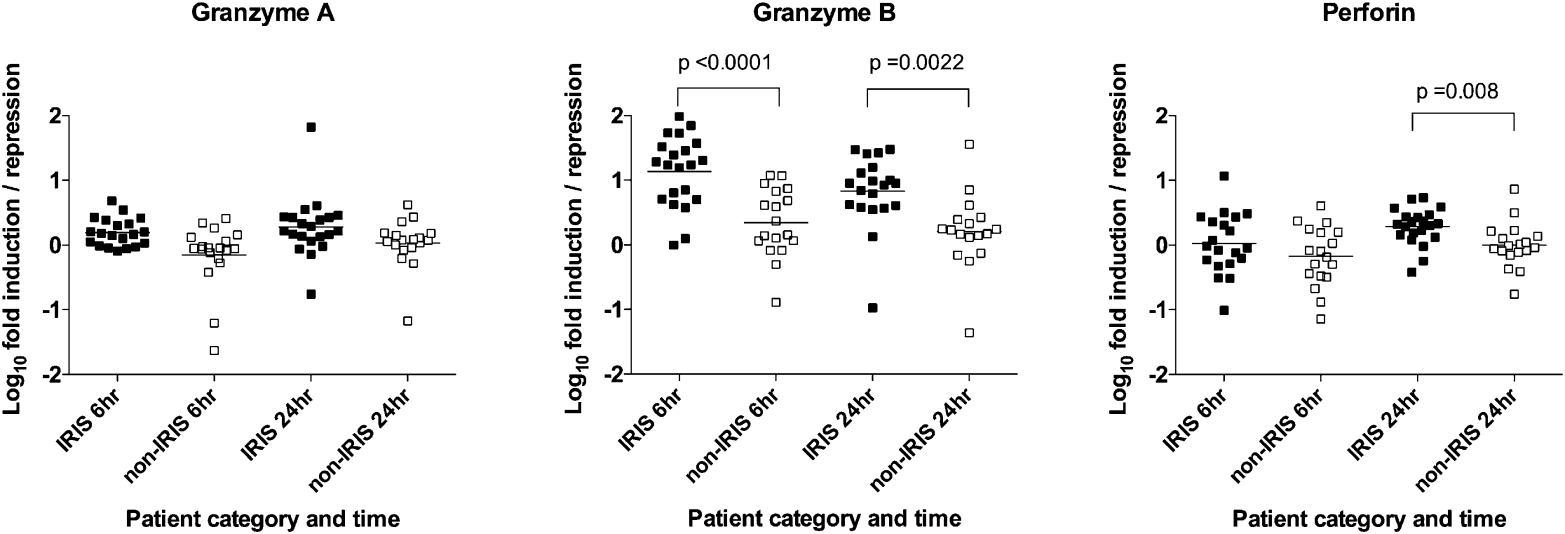
Secondary validation of microarray analysis by RT-PCR. PBMC from 22 TB-IRIS and 22 non-IRIS control patients were isolated and cultured in the presence or absence of hkH37Rv (MOI 1:1, PBMC:H37Rv) for 6 or 24 h. RNA was extracted and assayed by RT-PCR. β-actin was used to normalize values. Fold induction over unstimulated cultures was calculated by the ΔΔ cycle threshold method, and fold values log10 transformed to normalize. Granzyme B was increased in TB-IRIS compared with controls after both 6 and 24 h (*p* < 0.0001 and *p* = 0.002, respectively). Perforin was also significantly increased after 24-h stimulation (*p* = 0.008). Horizontal lines indicate median values.

To biologically and technically validate the microarray findings, quantitative RT-PCR analysis was performed in a second set of RNA samples from a larger number of 22 TB-IRIS and 22 non-IRIS control patients. The clinical characteristics of these patients are shown in Supplemental Table I and were previously reported (10). Freshly isolated PBMC at 2 wk post-ART initiation were cultured in the presence or absence of hkH37Rv for 6 or 24 h. Cell culture supernatants were collected at 24 h, whereas RNA was extracted at both time points and assayed, as described (10). Granzyme B transcript abundance was increased in TB-IRIS compared with non-IRIS controls after both 6 and 24 h (*p* < 0.0001 and *p* = 0.002, respectively; Fig. 3). Perforin transcript was also significantly more abundant after 24 h (*p* = 0.008). Granzyme A was included as an internal control and showed no difference in abundance between the groups at any time point.

**FIGURE 4.**
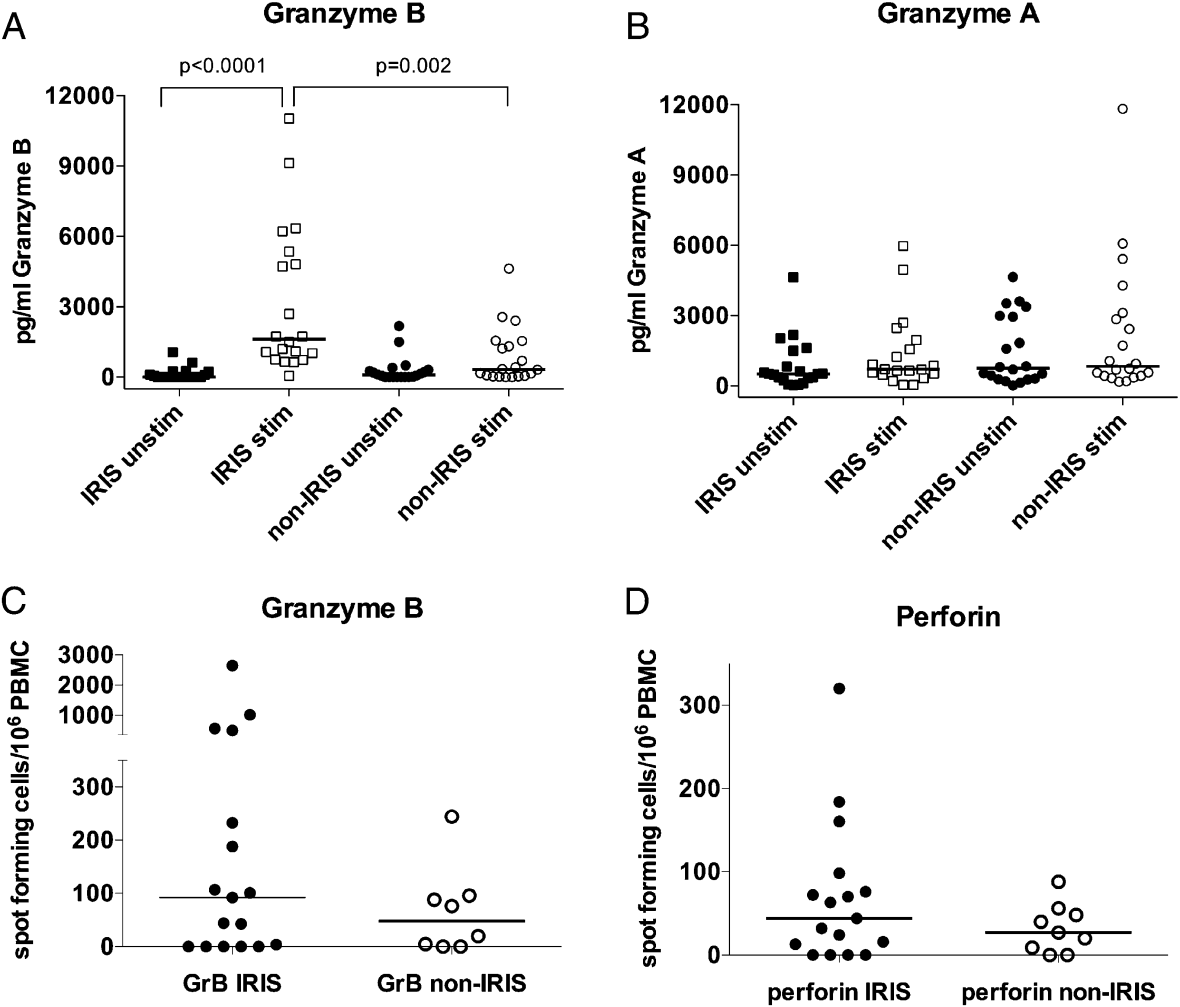
Granzyme B and perforin secretion are increased in TB-IRIS. (**A** and **B**) Granzyme B secretion measured by ELISA was significantly increased in TB-IRIS patients, in contrast to granzyme A, in hkH37Rv-stimulated PBMC culture supernatants (20 TB-IRIS, 20 controls) compared with unstimulated supernatants (*p* < 0.0001), and compared with stimulated PBMC culture supernatants from non-IRIS control patients (*p* = 0.002). (**C** and **D**) Granzyme B and perforin measured by ELISPOT tended to be increased in TB-IRIS compared with controls (not statistically significant). Horizontal lines indicate median values.

Next we evaluated granzyme A and B protein secretion in the PBMC culture supernatants collected at 24 h poststimulation with hkH37Rv in a subset of 20 TB-IRIS and 20 non-IRIS control patients, using ELISA. We found significantly increased granzyme B concentrations in TB-IRIS patients (median, 1617 pg/ml; IQR, 826.5–5227) compared with non-IRIS controls (median, 332.5 pg/ml; IQR, 42.50–1486; *p* = 0.002; Fig. 4A) and compared with unstimulated cultures. By contrast, granzyme A was not increased (Fig. 4B).

To confirm perforin secretion, we opted to employ the more sensitive ELISPOT methodology based on Zuber et al. (19), showing that the ELISPOT displayed greater detection sensitivity than the ELISA when assessing perforin release. Because granzyme A was consistently shown not to be elevated in our internal control experiments, we did not include it in the ELISPOT analysis, but we included measurement of granzyme B to further strengthen our findings. Thus, ELISPOT assay for perforin and granzyme B was performed in a smaller number of samples, based on stored PBMC availability. A nonsignificant trend toward increased numbers of PBMC-secreting granzyme B and perforin in response to hkH37Rv in TB-IRIS was observed (Fig. 4C, 4D). The median spot-forming cells per 10^6^ PBMC for granzyme B from TB-IRIS patients was 92 (IQR, 0–364) compared with 48 (IQR, 1.25–94) in non-IRIS patients. For perforin, the median from TB-IRIS patients was 44 (IQR, 6.5–87) in TB-IRIS and 27 (IQR, 4.5–52) in non-IRIS patients.

### The effect of in vivo prednisone therapy on granzyme B secretion in vitro

**FIGURE 5.**
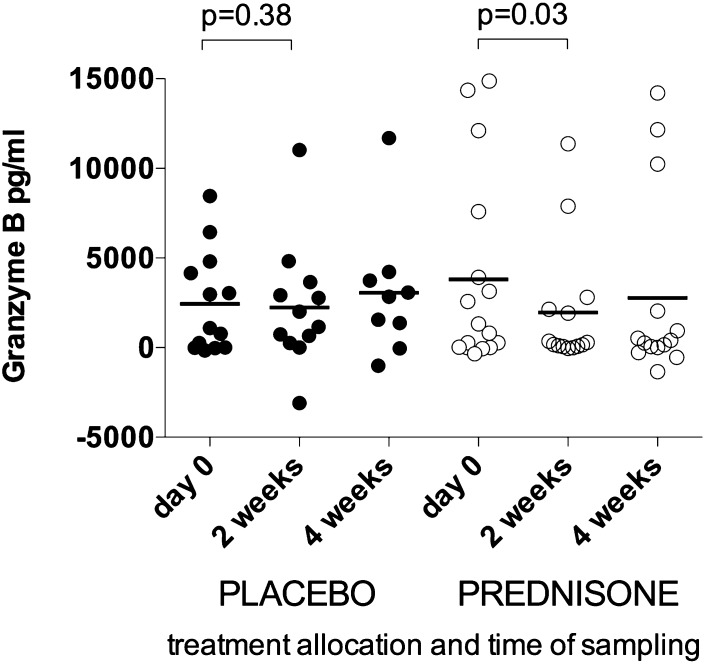
Effect of prednisone or placebo on granzyme B release in vitro. Granzyme B secretion (pg/ml) from PBMCs, from TB-IRIS patients enrolled in a randomized controlled trial of prednisone versus placebo, measured by ELISA after stimulation with hkH37Rv, with unstimulated culture results subtracted. Prednisone treatment for 2 wk significantly decreased granzyme B secretion from TB-IRIS PBMC compared with pretreatment values. This reduction was not evident in PBMC from placebo-treated TB-IRIS patients.

Corticosteroid therapy is used as an immunosuppressive therapy in the treatment of TB-IRIS. We evaluated granzyme B secretion by ELISA in the 24-h supernatant of hkH37Rv-stimulated PBMC cultures from a subset of 29 patients enrolled in a randomized double-blind placebo-controlled trial of prednisone for treatment of TB-IRIS (7, 8). The trial showed an overall reduction in duration of hospitalization and numbers of therapeutic procedures in prednisone-treated patients, as well as hastened improvement in TB-IRIS symptoms (7, 8), and such trends were evident in the subset of patients included in this analysis. Thus, we observed that in TB-IRIS patients receiving prednisone treatment for 2 wk, granzyme B secretion was decreased in vitro (*n* = 16; median, 233 pg/ml; IQR, 66–2312 pg/ml) compared with pretreatment responses (median, 1068; IQR, 6–6672; *p* = 0.03). This reduction was not evident in PBMC from placebo-treated patients (*n* = 13; Fig. 5), suggesting a correlation between our in vitro assessment of perforin and granzyme B and improved clinical outcome (7, 8).

### Elucidating the cellular source of perforin in TB-IRIS patients

Our findings suggested the differential induction of cytotoxic pathways in TB-IRIS in response to hkH37Rv. We therefore investigated potential cytotoxic cells present in PBMC at 2 wk post-ART initiation in a subgroup of patients using flow cytometry. As both CD8 and CD4 T cells are able to upregulate mRNA expression for granzyme B and perforin after stimulation with H37Rv (20), we first evaluated differences between these cells in TB-IRIS compared with non-IRIS controls. CD4 and CD8 T cells were present in similar proportions in unstimulated PBMC in TB-IRIS and non-IRIS controls (Supplemental Fig. 1A). In a subset of patients, we established that both CD4 and CD8 cells contained perforin (Supplemental Fig. 1B), which decreased on stimulation with hkH37Rv, implying Ag-specific degranulation (as shown in a smaller subset of patients in Supplemental Table II). Next, we quantified NK cell proportions, identified by the absence of CD3, the presence of CD16, and a combination of activating (CD56) or inhibitory (CD94, CD158) cell surface receptors. No difference in NK cell proportions was observed in TB-IRIS, compared with non-IRIS controls (Supplemental Table III). The γ-δ (γδ) T cells have been implicated in TB-IRIS pathophysiology, can recognize nonprotein Ags, and express perforin and granzyme B (21, 22). We observed a trend toward decreased proportions of γδ TCR-expressing cells in TB-IRIS compared with non-IRIS PBMC (Supplemental Table III; *p* = 0.089), suggesting that this population is less likely to contribute to upregulation of the cytotoxic pathways identified.

**FIGURE 6.**
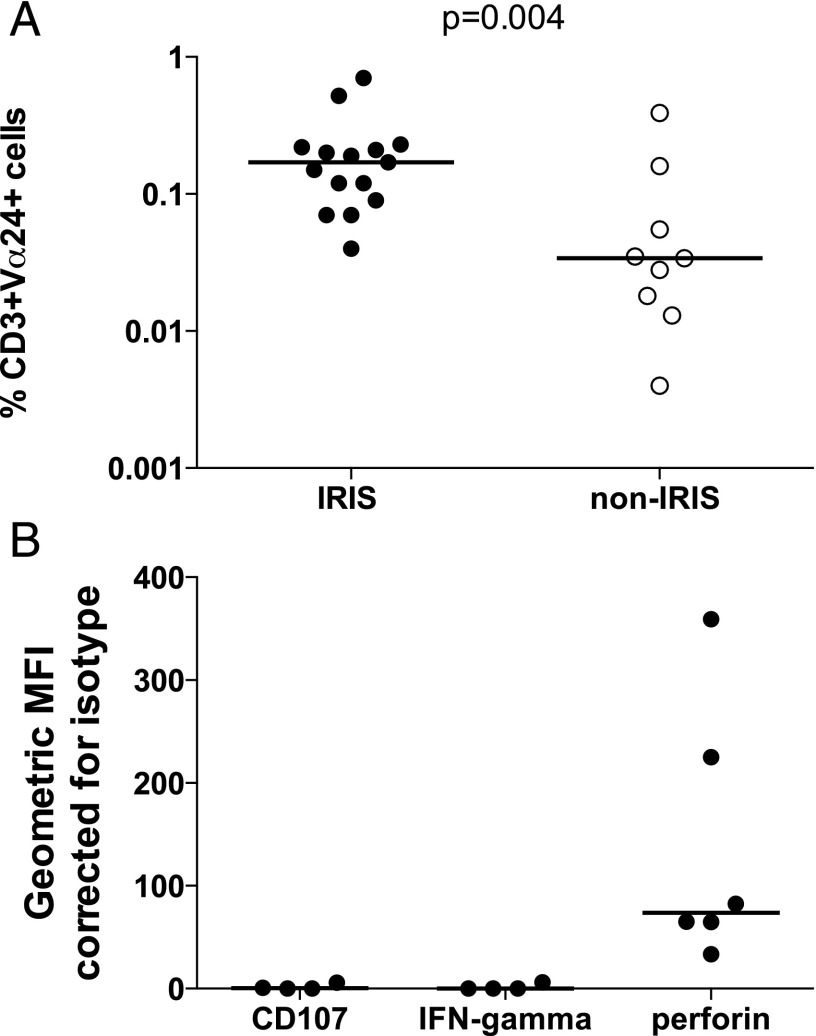
Increased proportions of iNKT cells in TB-IRIS compared with non-IRIS control patients. Flow cytometric analysis using cell surface staining for CD3^+^Vα24^+^ cells identified an increased proportion of CD3^+^Vα24^+^ cells in TB-IRIS compared with non-IRIS control patients (median, 0.17%; IQR, 0.09–0.22, versus 0.03%; IQR, 0.016–0.106) (**A**). Using intracellular staining for perforin, CD3^+^Vα24^+^ cells from TB-IRIS patients were identified as a potential source of perforin (**B**). Horizontal bars indicate median values. Gating strategy was based on selecting CD3^+^ cells from a side scatter/CD3 scatter dot blot, followed by gating on Vα24 and either Vβ11 or perforin-positive cells.

iNKT cells may express either CD4 and/or CD8 molecules, are innate lymphocytes with cytotoxic activity, and are characterized by reactivity to glycolipid Ags. We investigated proportions of iNKT cells, as determined by the presence of Vα24 TCR that, in combination with Vβ11, characterizes these cells in humans. Significantly increased proportions of CD3^+^ Vα24^+^ cells were found in unstimulated PBMC at 2 wk post-ART initiation from TB-IRIS patients: median, 0.17% (IQR, 0.09–0.22; *n* = 15), compared with non-IRIS controls (median, 0.03%; IQR, 0.016–0.106; *n* = 9; *p* = 0.004; Fig. 6A). Using the more stringent combination of Vα24^+^Vβ11^+^ staining on CD3^+^ cells in a smaller number of TB-IRIS patients (*n* = 11), the median CD3^+^Vα24^+^Vβ11^+^ frequency was 0.18% (IQR, 0.09–0.4) compared with 0.04% (IQR, 0.03–0.82; *n* = 9) in nine non-IRIS patients (*p* = 0.05; Supplemental Fig. 1C). Moreover, intracellular staining in a subset of these TB-IRIS patient samples identified these cells as a source of perforin (Fig. 6B).

## Discussion

Defining the pathophysiology of TB-IRIS may facilitate development of diagnostic tests and specific treatments, which are currently lacking. Although dysregulated immune restoration is associated with dynamic CD4 Th1 expansions and contractions, these relate poorly to symptoms (8, 9). In our previous longitudinal analysis, we found no evidence for a difference between TB-IRIS and non-IRIS patients in response to recombinant protein Ags and purified protein derivative, suggesting that protein Ag-specific Th1 responses may not be the primary determinants of TB-IRIS. The finding in our current study, that increased IFN-γ responses occur in response to hkH37Rv in TB-IRIS patients at TB-IRIS onset compared with non-IRIS controls, raises the possibility that a component of the T cell response directed toward nonprotein Ags may be responsible for the differential response.

Our microarray analysis, demonstrating perforin 1 and granzyme B to be the most significantly upregulated genes in PBMC from TB-IRIS patients, compared with non-IRIS controls, and further validation of these results with RT-PCR, ELISA, and ELISPOT, highlights a role for the granule–exocytosis pathway in TB-IRIS. Perforin is a cytolytic protein found in granules of innate lymphocytes (cytotoxic T lymphocytes and NK cells) and neutrophils that have a key role in delivery of contents of cytotoxic granules to target cell cytoplasm. Granzyme B is a serine protease, a key component of cytotoxic granules, and activates apoptosis once in the cytoplasm of the target cell. Potential cell types that are capable of recognizing nonprotein Ags have cytotoxic capacity and contain granule-associated perforin and granzyme B, including NK cells, γδ T cells, and NKT cells (21, 23, 24). Two recent studies have examined the potential contribution of NK cells in TB-IRIS and suggested that elevated NK cell activation and degranulation levels characterize the immunological profile of TB-IRIS patients (25, 26). One previous study also demonstrated an association between a subset of γδ T cells and TB-IRIS (22). However, we found reduced proportions of these cells in TB-IRIS, suggesting that they cannot be responsible for the increased perforin and granzyme B detected in TB-IRIS patients. We demonstrate that both CD4 and CD8 cells were a source of perforin in TB-IRIS patients and that increased proportions of iNKT cells are present in PBMC of TB-IRIS patients at the time of IRIS onset, compared with non-IRIS controls, in two analyses (calculating CD3^+^Vα24^+^ cells and CD3^+^Vα24^+^Vβ11^+^ cells), both considered to be stringent approaches for quantifying iNKT cells (27). Although these results implicate iNKT cells as a likely candidate, we are currently conducting a longitudinal study addressing further the role of cytotoxic T cells in TB-IRIS development.

We have previously demonstrated that TB-IRIS is associated with hypercytokinaemia in blood and cerebrospinal fluid, of predominantly myeloid or dual myeloid/lymphoid in origin, which is modulated during treatment of TB-IRIS with corticosteroids (10, 12, 28). These and other studies exploring murine models of TB-IRIS have led to the recent proposal that ART-induced changes in innate immune function contribute substantially to TB-IRIS (11, 28, 29). Our observation that granzyme B gene upregulation and protein secretion occur in PBMC from patients at the time of TB-IRIS symptom onset, and that secretion is reduced in PBMC from patients treated for TB-IRIS by prednisone, which improves symptoms and reduces hospitalization, supports a role for cytotoxic mediators in the immunopathology of TB-IRIS. Further characterization of this cytotoxic response, especially at the site of infection, is desirable.

Our study has a number of limitations, including the limited number of cells available for in-depth analysis of cytotoxic lymphocytes. Inclusion of samples based on availability of PBMC means that some of the assays were performed in samples from different patients, possibly resulting in the introduction of selection bias. However, it is encouraging to find the same pattern of mediators in different samples collected over time. Our study design did not include the evaluation of nonprotein preparations such as lipid fractions, and we acknowledge the fact that, whereas killed mycobacteria will no longer secrete protein Ags, they still contain antigenic proteins, some of which will give rise to an immune response. Additionally, we did not assess the capacity of neutrophils to produce cytotoxic mediators. Evaluating the role of the two additional genes that were upregulated in TB-IRIS (ZNRF1: Zinc and ring finger 1 and CITED2: Cbp/p300-interacting transactivator with Glu/Asp-rich C-terminal domain 2) was outside the scope of the current study and should constitute the subject of further investigation.

In conclusion, our data indicate differential cytotoxic activity associated with TB-IRIS, with increased cytotoxic mediators compared with non-IRIS patients. This supports further analysis of cytotoxic pathways, not only in TB-IRIS pathophysiology, but also in the pathogenesis of tuberculosis and inflammatory conditions in general. Improved understanding of the mechanisms of pathology in TB-IRIS is required to derive new treatments to reduce the morbidity and mortality associated with this condition.

## Supplementary Material

Data Supplement
