# Supplementary material for: Cytotoxic Mediators in Paradoxical HIV–Tuberculosis Immune Reconstitution Inflammatory Syndrome
Source: J Immunol. 2015 Jan 14;194(4):1748–54. doi: 10.4049/jimmunol.1402105 (PMC4319311; doi:10.4049/jimmunol.1402105)
Supplement: Data Supplement [file JI_1402105.zip › JI_1402105_Supplemental_Material_1.pdf]

Supplementary Information

Cytotoxic mediators in paradoxical HIV-tuberculosis immune reconstitution inflammatory syndrome[1,2]

Katalin A Wilkinson[*,†,¶,#], Naomi F Walker[*,‡,#], Graeme Meintjes[*,†,‡], Armin Deffur[*], Mark P Nicol[*,||], Keira H Skolimowska[*,‡], Kerryn Matthews[*], Rebecca Tadokera[*], Ronnett Seldon[*], Gary Maartens[†], Molebogeng X Rangaka[*], Gurdyal S Besra[§], Robert J Wilkinson[‡,*,†,¶]

[*]Clinical Infectious Diseases Research Initiative and [†]Department of Medicine, University of Cape Town, South Africa; [‡]Division of Medicine, Imperial College London, W2 1PG, UK; [§]School of Biosciences, University of Birmingham, UK; [¶]MRC National Institute for Medical Research, London, NW7 1AA, UK; [||]Division of Medical Microbiology, University of Cape Town and National Health Laboratory Service, South Africa.

Supplementary Figure1:	Elucidating the cellular source of perforin in TB-IRIS patients
Supplementary Table 1:	All patients, clinical information
Supplementary Table 2:	Perforin decreases on stimulation with heat-killed H37Rv
Supplementary Table 3:	γδ T cells and Natural Killer (NK) cells in TB-IRIS compared to non-IRIS patients using flow cytometry.

[1] All authors declare no competing interests.
Preliminary data was reported at the Host Response in Tuberculosis Keystone Symposia (March 2013, poster X7 4041).

[2] Funding: this work was supported by the Wellcome Trust [grant numbers 081667, 084323, 088316, 094000, 085251]; the Medical Research Council of the United Kingdom [grant number U.1175.02.002.00014.01]; the European and Developing Countries Clinical Trials Partnership [grant number IP.07.32080.002]; and the European Union [PIRSES-GA-2011-295214; FP7-Health-F3-2012-305578].

Supplementary Figure 1: Elucidating the cellular source of perforin in TB-IRIS patients

(A) Flow cytometric assessment demonstrated that CD4 and CD8 cells are present in similar proportions in unstimulated PBMC in TB-IRIS and controls;
(B) both cell types contain perforin. Statistical tests showed no significant difference in CD4 and CD8 cell proportions and in perforin expression between TB-IRIS and non-IRIS controls. Horizontal bars indicate median values. MFI: mean fluorescence index.
(C) Flow cytometric analysis of peripheral blood mononuclear cells from 11 TB-IRIS patients and 9 non-IRIS control patients, using cell surface staining for CD3+Vα24+Vβ11+ cells identified an increased proportion of CD3+Vα24+Vβ11+ cells in TB-IRIS compared to non-IRIS control patients, although this did not reach statistical significance (median 0.18%, IQR 0.09-0.4, vs 0.04%, IQR 0.03-0.82, p=0.05). Horizontal bars indicate median values.

A

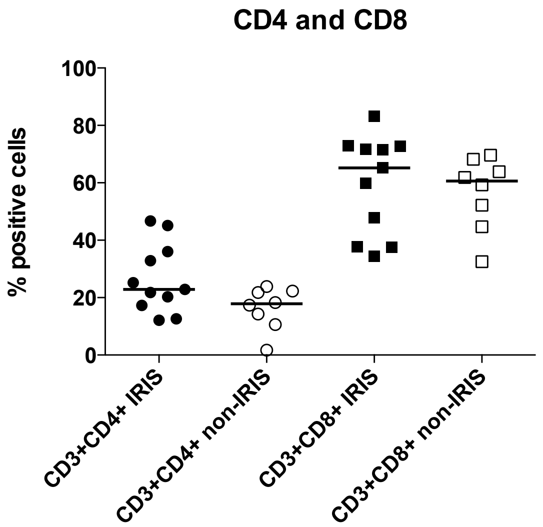

B

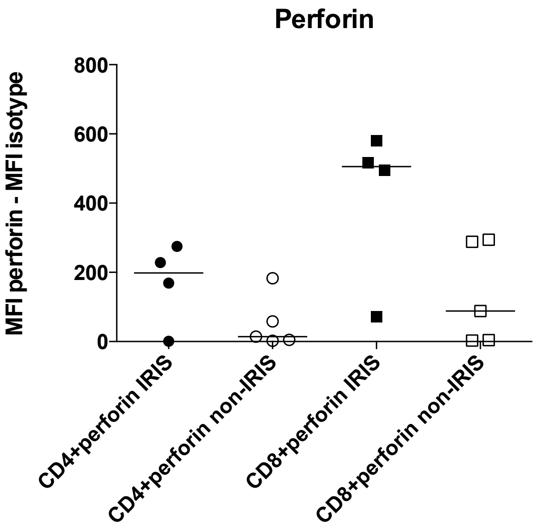

C

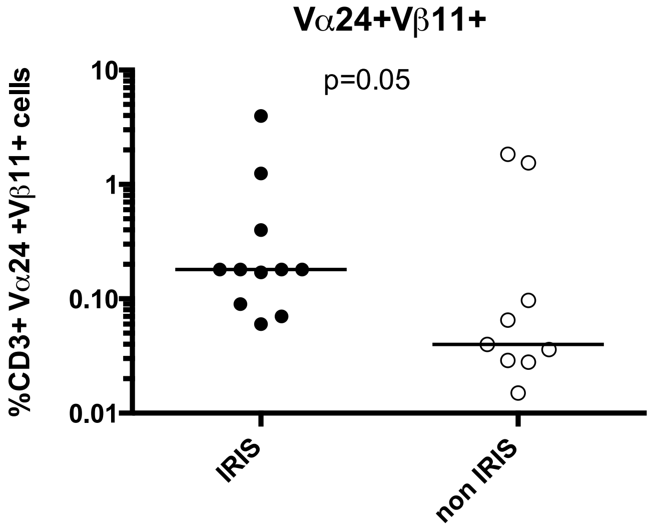

Supplementary Table 1. All patients, clinical information

IRIS number	Gender	Age	Baseline CD4 Count	TB Disease form	Microbiological confirmation	WHO stage at ART initiation	Experiment	Time to IRIS onset	IRIS Details	RCT allo-cation
3	Female	56	65	Miliary TB	Yes	4	5	14	Systemic, pulmonary, nodal	n/a
9	Female	28	366	Pulmonary	Yes	3	5	10	Systemic, pulmonary, abdominal	n/a
10	Female	24	94	Pleuropulmonary	Yes	3	5	14	Pulmonary, nodal	n/a
13B	Female	37	92	Pulmonary	Yes	3	1	13	Lymphadenitis	n/a
13	Female	29	103	Cervical lymphadenitis	Yes	4	5	46	Systemic, nodal	n/a
16	Male	27	20	Miliary, lymphadenopathy	No	4	5	60	CNS (Meningitis)	n/a
17	Male	46	108	Pulmonary	Yes	4	5	42	Pulmonary, nodal, pericardial	n/a
18	Male	25	166	Pleural effusion	Yes	4	3,4	55	Pulmonary, nodal	n/a
20	Male	36	92	Pulmonary	No	3	1	28	Pulmonary IRIS	n/a
21	Female	40	193	Pericardial effusion	No	4	3,4,5	18	Systemic, pulmonary, abdominal	n/a
22	Female	35	153	Pulmonary	Yes	3	2,3,4, 5,6	10	Pulmonary, nodal, pericardial, abdominal	Placebo
24	Female	23	56	Disseminated, including CNS	Yes	4	2,3,4	24	Pulmonary, nodal, abdominal, CNS	n/a
26	Male	34	160	Disseminated	Yes	4	5	14	Nodal, abdominal	n/a
28	Male	45	30	Disseminated tuberculosis	No	4	1	7	Pulmonary IRIS	n/a
30	Female	42	42	Pulmonary	No	3	1	14	Lymphadenitis	n/a
31	Female	37	71	Disseminated tuberculosis	Yes	4	1	13	Abdominal IRIS	n/a
33	Male	28	5	Pleural effusion	Yes	4	1	14	Pulmonary IRIS	n/a
34B	Male	29	13	Pulmonary	Yes	3	1	15	Pulmonary IRIS	n/a
34	Male	30	79	Cervical lymphadenitis	No	3	3,4,6	9	Nodal	Placebo
38	Male	36	10	Disseminated tuberculosis, with meningitis	Yes	3	1	7	Neurological and abdominal IRIS	n/a
41	Female	23	55	Disseminated, with meningitis	Yes	4	3,4,5	78	Pulmonary, nodal, abdominal	n/a
42	Female	29	67	Pulmonary	Yes	4	3,4,6	37	Pulmonary, nodal, abdominal	Prednisone
43	Female	23	158	Pulmonary	Yes	3	4,4	17	Pulmonary, abdominal	n/a
44	Female	51	49	Pulmonary	Yes	3	2,3,6	17	Systemic, pulmonary, abdominal	Prednisone

47	Male	40	30	Disseminated	Yes	4	3,4,6	13	Pulmonary, abdominal	Prednisone
48	Female	32	36	Pleuroperitoneal	Yes	4	2,6	3	Pulmonary, nodal, abdominal	Placebo
49B	Female	36	113	Disseminated tuberculosis	Yes	4	1	12	Neurological IRIS	n/a
49	Female	51	106	Axillary lymphadenitis	Yes	4	3,4	14	Nodal	n/a
50	Female	26	48	Pulmonary, cervical lymphadenopathy	Yes	3	1,5		Abdominal IRIS	n/a
51	Female	31	1	Pulmonary	Yes	4	5	180	Nodal, thyroid mass	n/a
52	Female	20	48	Disseminated tuberculosis	No	4	1	28	Lymphadenitis	n/a
53	Female	34	38	Disseminated, with pericardial involvement	Yes	4	3,4	44	Nodal, abdominal, cold abscess on forearm	n/a
55	Male	33	13	Pulmonary	Yes	4	6	8	Pulmonary, nodal, abdominal	Placebo
62	Male	36	39	Pulmonary, cervical lymphadenitis	Yes	4	3,4,6	14	Pulmonary, nodal, abdominal	Prednisone
70	Male	37	25	Pulmonary	Yes	3	6	7	Pulmonary	Prednisone
71	Female	25	68	Pulmonary	Yes	3	3,5	14	Pulmonary, nodal	n/a
72	Male	45	46	Pulmonary	Yes	3	3,4,6	6	Pulmonary, nodal	Prednisone
73	Female	23	174	Pulmonary	Yes	3	3,4	13	Nodal, abdominal	n/a
77	Male	24	14	Pulmonary	Yes	3	3,4	7	Pulmonary, nodal, abdominal	n/a
83	Female	32	37	Disseminated, with pleural effusion	Yes	4	3,4,6	21	Pulmonary, nodal, abdominal	Prednisone
90	Male	24	47	Disseminated	Yes	3	6	29	Pulmonary, nodal, abdominal	Prednisone
93	Male	25	181	Pulmonary	Yes	3	5,6	14	Pulmonary, nodal, abdominal	Prednisone
94	Female	19	84	Disseminated, with pericardial involvement	Yes	4	6	17	Pulmonary, nodal, abdominal	Placebo
97	Female	24	156	Pulmonary	Yes	3	6	14	Nodal	Placebo
98	Female	31	7	Pulmonary	No	3	2	14	Pulmonary, nodal	n/a
105	Female	27	103	Pulmonary	Yes	4	5,6	13	Pulmonary, nodal, abdominal	Placebo
107	Female	28	51	Pulmonary	Yes	3	3,4,6	7	Pulmonary	Prednisone
110	Female	30	24	Disseminated, with pericardial involvement	Yes	4	6	5	Pulmonary, nodal, abdominal	Placebo
113	Male	36	95	Pulmonary	Yes	3	2,3,4,6	5	Systemic, pulmonary, abdominal	Placebo
116	Male	57	29	Miliary TB	Yes	4	2	14	Systemic, pulmonary	n/a
118	Female	30	99	Pulmonary	Yes	4	2,3,4,6	10	Pulmonary, abdominal	Prednisone

121	Female	21	226	Pulmonary	Yes	3	5	10	Pulmonary	n/a
129	Female	33	48	Disseminated	No	4	3,4	21	Systemic, nodal, abdominal	n/a
141	Male	33	150	Disseminated, with pericardial involvement	Yes	4	6	25	Pulmonary, abdominal	Placebo
192	Male	39	58	Pulmonary	Yes	3	6	7	Pulmonary	Placebo
206	Male	24	5	Pulmonary	Yes	4	6	7	Pulmonary, nodal	Placebo
215	Female	23	21	Pulmonary	Yes	3	6	14	Pulmonary	Prednisone
224	Female	34	167	Lymphadenitis	No	4	6	14	Pulmonary, nodal, abdominal	Prednisone
230	Male	27	55	Pulmonary	Yes	3	5	14	Abdominal	n/a
236	Female	32	68	Pulmonary	No	3	6	19	Pulmonary, abdominal	Prednisone
238	Female	27	13	Pulmonary	Yes	3	6	8	Pulmonary, nodal	Prednisone
247	Female	30	78	Disseminated, with pleural effusion	Yes	4	6	17	Pulmonary	Prednisone
257	Male	34	26	Disseminated	Yes	4	6	7	Pulmonary, nodal, abdominal, CNS	Placebo

**non-IRIS nr**	**Gender**	**Age**	**Baseline CD4 Count**	**TB Disease form**	**Microbiological confirmation**	**WHO Stage at HAART Start**	**Experiment**
0	Female	22	120	Pulmonary	No	3	1,2,3,4
1	Female	42	170	Pleural	No	4	1
2	Female	33	90	Pulmonary	No	3	5
3	Female	24	215	Lymphadenitis	Yes	4	1
4	Male	32	18	Pulmonary	Yes	4	1
7	Female	30	115	Lymphadentitis and pleural effusion	Yes	4	1
11	Female	39	302	Mediastinal adenitis	No	3	1,3,4
12	Male	35	159	Pulmonary	Yes	3	3,4
14	Female	48	172	Pulmonary, lymphadenitis	Yes	4	3,4
15	Female	29	186	Pulmonary	No	3	2,3
16	Female	27	40	Pulmonary, pleural	Yes	4	3,4
24	Female	44	40	Pulmonary	Yes	3	3,4
26	Female	31	27	Pulmonary, pleural	Yes	4	1,4
27	Male	35	38	Pulmonary	No	3	1,2,3,4
32	Female	41	29	Disseminated tuberculosis	No	4	1,2,3,4
35	Female	36	18	Pleural	Yes	4	1,2,3,4
36	Female	37	139	Pulmonary	No	3	1,2,5
37	Female	46	45	Pulmonary	Yes	3	1,2,3,4
39	Male	48	28	Pleural	No	4	1,3,4,5
40	Female	30	19	Pulmonary	Yes	4	1,3,4,5
41	Female	26	19	Pulmonary	Yes	4	3,4
42	Female	38	37	Peritoneal	Yes	4	1
44	Female	26	48	Pulmonary	Yes	3	1,3,4,5

45	Male	40	5	Pulmonary, Extrapulmonary	Yes	4	1,3,4
47	Male	34	123	Pulmonary	Yes	3	1,3,4
51	Female	33	31	Disseminated tuberculosis	Yes	3	1,5
54	Male	30	121	Pulmonary	No	4	1
55	Male	44	7	Pulmonary	No	4	1,3,5
56	Male	35	51	Pulmonary	Yes	3	1,3,4, 5
59	Female	27	20	Disseminated tuberculosis, with meningitis	No	4	1,3,5
60	Female	54	99	Pleural effusion	No	3	1,3,4
62	Female	22	68	Pleural effusion	No	4	1,3,4, 5
63	Female	21	149	Pulmonary	No	3	1
64	Female	37	96	Pulmonary	No	3	1

*Key for experiment: (1) Figure 1; (2) Figure 2; (3) Figure 3; (4) Figure 4A,B; (5) Figure 4C,D; (6) Figure 5. n/a: not applicable

Supplementary Table 2.

Perforin decreases on stimulation with heat-killed H37Rv. In a subset of one non-IRIS and two TB-IRIS patients we established that perforin decreases on stimulation with heat-killed H37Rv in both CD4 and CD8 T cell subsets, suggesting antigen-specific degranulation. No effect on antigen stimulation was seen in the presence of Isotype control staining. MFI: mean fluorescence index. ND: not done.

	Perforin staining (MFI)				Isotype control (MFI)			
Sample	Unstimulated		Stimulated		Unstimulated		Stimulated	
	CD4	CD8	CD4	CD8	CD4	CD8	CD4	CD8
IRIS 1	249	614	144	324	ND	ND	66.2	77.2
IRIS 2	315.4	659.7	241.6	577.4	87.1	79.1	76.5	72.6
Non-IRIS	136.8	394.8	98.1	258.5	77.2	72.9	75.3	67.7

Supplementary Table 3.

γδ T cells and Natural Killer (NK) cells in TB-IRIS compared to non-IRIS patients using flow cytometry. Flow cytometric analysis of peripheral blood mononuclear cells (PBMC) from TB-IRIS patients compared with non-IRIS controls. Values are expressed as median % of total PBMC (with interquartile range). Numbers of patients studied are given in square brackets [n].

Cell Type	IRIS [n]	Non-IRIS [n]	p
γδTCR+CD3+	4.6 (3.23-6.83) [12]	10.3 (3.2-14) [7]	0.089
NK CD3- CD56+CD16+	20.1 (11.9-56) [9]	29.3 (11.5-49.1) [5]	0.937
NK CD3- CD94+	56.3 (37.7-65.9) [8]	45.2 (27-62.2) [7]	0.387
NK CD3- CD158+	15.3 (9.3-26.2) [8]	36.6 (9.8-78.2) [7]	0.151
